# Cutaneous Malignancy Risk in Facial and Hand Vascularized Composite Allotransplantation Recipients: A Review of Immunosuppressive Regimens and Their Oncologic Impact

**DOI:** 10.3390/life16040544

**Published:** 2026-03-25

**Authors:** Beatrice Corsini, Ferruccio Paganini, Sara Matarazzo, Luigi Valdatta

**Affiliations:** 1 Division of Plastic and Reconstructive Surgery, Department of Biotechnology and Life Sciences, University of Insubria, 21100 Varese, Italy; fpaganini@uninsubria.it (F.P.); smatarazzo@studenti.uninsubria.it (S.M.); 2 Department of Medicine and Surgery, University of Insubria, 21100, Varese, Italy

**Keywords:** facial transplantation, vascularized composite allotransplantation (VCA), immunosuppressive therapy, cutaneous malignancy, non-melanoma skin cancer

## Abstract

Facial vascularized composite allotransplantation (fVCA) is one of the most complex forms of vascularized composite allotransplantation and requires lifelong immunosuppression to ensure graft survival. Despite significant advances in surgical techniques and postoperative care over the past two decades, the true incidence of cutaneous malignancies in fVCA recipients remains poorly characterized due to the limited number of procedures, heterogeneous immunosuppressive protocols, and relatively short follow-up. This narrative review summarizes current evidence on oncologic risk in facial VCA, focusing on the effects of different immunosuppressive regimens and the challenges posed by the high immunogenicity of skin and mucosa. Available data indicate that malignancies, including cutaneous and other neoplasms, occur in approximately 10% of recipients, based on heterogeneous case-series data with immunosuppressive therapies largely extrapolated from solid organ transplantation. Calcineurin inhibitors, corticosteroids, and azathioprine are associated with increased oncologic risk, whereas mycophenolate mofetil and mTOR inhibitors may confer a more favorable profile. Overall, fVCA, unlike solid organ transplantation, is a life-enhancing procedure, highlighting the need for tailored immunosuppressive strategies, rigorous dermatologic surveillance, and further research supported by dedicated registries to better define long-term malignancy risk.

## 1. Introduction

Since 2005, approximately 50 facial transplantation procedures have been documented worldwide, each involving different combinations of aesthetic units and facial structures [1]. While several institutions now consider facial transplantation an established therapeutic option, others continue to carry it out within experimental or investigational frameworks [2].

Advances in transplant medicine have significantly improved long-term survival and quality of life for patients undergoing organ replacement therapies; however, lifelong immunosuppression remains essential to prevent graft rejection and is associated with a substantially increased risk of malignancy [3]. Cutaneous malignancies represent the most frequent post-transplant cancers, accounting for more than 90% of all malignancies in transplant recipients [4]. In particular, non-melanoma skin cancers (NMSCs), including cutaneous squamous cell carcinoma (cSCC) and basal cell carcinoma (BCC), occur at markedly higher rates than in the general population and often exhibit more aggressive clinical behavior [5]. The development of post-transplant skin cancer reflects a multifactorial process involving impaired immune surveillance, ultraviolet radiation exposure, oncogenic viral infections, and the pro-tumorigenic effects of specific immunosuppressive agents [6]. In solid organ transplantation, both the dose and duration of immunosuppression are well-established determinants of cancer risk [7,8,9], with calcineurin inhibitors, azathioprine, and corticosteroids being associated with increased oncologic burden, whereas mycophenolate mofetil and mammalian target of rapamycin (mTOR) inhibitors appear to confer a more favorable oncologic profile.

Facial transplantation is one of the most complex forms of vascularized composite allotransplantation (VCA), involving the transfer of multiple highly immunogenic tissues, including skin and mucosa. In this context, hand transplants can also be considered different from other solid organ transplants because they contain bone marrow, which has the potential to induce donor-specific tolerance through the establishment of mixed chimerism [10,11]. 

Unlike solid organ transplantation, facial VCA (fVCA) is a life-enhancing rather than life-saving procedure [12], raising ethical and clinical concerns regarding the long-term consequences of lifelong immunosuppression, particularly the risk of malignancy, in an otherwise healthy population [10,13]. Despite significant advances in surgical techniques and postoperative care over the past two decades, the true incidence of cutaneous malignancies in fVCA recipients remains poorly characterized.

This uncertainty is mainly due to the limited number of procedures, heterogeneous immunosuppressive protocols, and relatively short follow-up.

Whether oncologic risks observed in solid organ transplantation can be directly extrapolated to fVCA remains uncertain, especially in the context of emerging immunosuppression-minimization strategies [13,14].

The aim of this narrative review is to summarize current evidence on the risk of cutaneous malignancies in facial transplant recipients, with a specific focus on the oncologic implications of different immunosuppressive regimens, highlighting existing knowledge gaps and the need for tailored, oncology-aware immunosuppressive strategies and long-term dermatologic surveillance in this highly selected patient population.

## 2. Materials and Methods

This study was designed as a structured narrative review rather than a systematic review. The aim was to provide a comprehensive synthesis of the available literature on the risk of cutaneous malignancies associated with immunosuppressive therapy in transplant recipients, with particular focus on facial and hand vascularized composite allotransplantation (VCA).

PubMed, Scopus, and Web of Science databases were searched for articles published from 2005 to 2025.

Studies were considered eligible if they reported data on the incidence, risk factors, or mechanisms of cutaneous malignancies in transplant recipients, or if they described immunosuppressive regimens used in vascularized composite allotransplantation or solid organ transplantation. Case reports, case series, clinical studies, and relevant narrative or systematic reviews were included to allow a broad overview of the available evidence.

Articles were excluded if they did not address oncologic outcomes, immunosuppressive regimens, or dermatologic complications in transplant recipients. Studies focusing on unrelated dermatologic conditions or lacking sufficient clinical information were also excluded.

Search terms included “facial transplantation,” “hand transplantation,” “vascularized composite allotransplantation,” “solid organ transplantation,” “immunosuppressive therapy,” “cutaneous malignancies,” “non-melanoma skin cancer,” and “squamous cell carcinoma.” Additional terms such as “basal cell carcinoma,” “mTOR inhibitors,” and “calcineurin inhibitors” were also included to capture relevant studies on immunosuppressive regimens and their oncologic impact.

To broaden the available evidence base, the search strategy was expanded to include studies on hand transplantation in addition to facial transplantation. This methodological choice was based on the comparable antigenicity of these two forms of vascularized composite allotransplantation (VCA), both of which involve highly immunogenic tissues such as skin, subcutaneous tissue, muscle, nerves, and vascular structures. As a result, the immunosuppressive regimens employed and the underlying immunologic mechanisms are largely overlapping, making data from hand transplantation relevant when assessing cutaneous oncologic risk in facial VCA recipients.

However, this approach does not imply that facial and hand transplantation carry identical oncologic risks. Rather, hand transplantation studies were included to complement the limited number of facial VCA cases and to explore whether differences in malignancy incidence might emerge between these forms of VCA.

Included articles were also analyzed with specific attention to the immunosuppressive regimens used for both induction and maintenance therapy. Particular emphasis was placed on long-term complications, including the development of secondary malignancies and their potential association with type, intensity, and cumulative exposure to immunosuppressive therapy. In addition, the articles included in this review were specifically analyzed with regard to the immunosuppressive regimens employed for both induction and maintenance therapy. Particular attention was given to long-term complication rates, including the development of secondary malignancies, as well as to the strength of the association between the late onset of de novo neoplasms and the type, intensity, and cumulative dose of immunosuppressive therapy administered.

Articles were initially screened for relevance based on title and abstract, followed by full-text evaluation for inclusion. Studies reporting the incidence, risk factors, or pathophysiologic mechanisms of skin cancer in transplant recipients, as well as clinical immunosuppressive protocols in both solid organ transplantation and facial or hand VCA, were included. Reviews, case series, and clinical reports were considered to provide a broad synthesis of existing knowledge. No language restrictions were applied, and reference lists of included articles were manually screened to identify additional pertinent publications.

This approach enabled the integration of epidemiologic data, immunosuppressive strategies, and mechanistic insights, providing a comprehensive overview of cutaneous malignancy risk in transplant populations and the oncologic implications of different immunosuppressive regimens. 

## 3. Results

After application of the eligibility criteria and full-text screening, a total of 18 articles were included in this narrative review. These studies consisted of case reports, case series, and reviews addressing immunosuppressive regimens and oncologic outcomes in vascularized composite allotransplantation and solid organ transplantation.

Because of the limited number of reported VCA procedures and the relatively short follow-up available, much of the evidence regarding malignancy risk and the oncologic effects of immunosuppressive therapy derives from studies in solid organ transplantation, particularly kidney transplantation. In the following sections, data originating from solid organ transplantation are presented separately from the limited evidence currently available in facial and hand VCA.

### 3.1. Epidemiology of Cutaneous Malignancies in Transplant Recipients in Solid Organ Transplantation

#### 3.1.1. Non-Melanoma Skin Cancer (NMSC)

NMSCs represent the majority of post-transplant skin malignancies, accounting for over 90% of cases [4,15,16,17]. Among these, SCC is particularly prevalent, occurring 65–250 times more frequently than in the general population and exhibiting a more aggressive clinical course. BCC is also increased, by approximately 10–20 times compared to immunocompetent individuals [18]. Notably, the typical ratio of SCC to BCC observed in the general population (~1:4) is reversed in transplant recipients (~4:1) [5].

#### 3.1.2. Cutaneous Manifestation

In addition to non-melanoma skin cancers, two further tumor types may arise in immunosuppressed individuals: Kaposi sarcoma, associated with human herpesvirus 8 (HHV-8), typically occurring 2–5 years after transplantation, and Merkel cell carcinoma, linked to Merkel cell polyomavirus [19].

Finally, transplant recipients may develop a broad spectrum of non-neoplastic cutaneous manifestations, including acneiform eruptions, non-scarring alopecia, lymphedema, gingival hyperplasia, hypertrichosis, and sebaceous hyperplasia [20].

#### 3.1.3. Risk Factors

**Figure 1:**
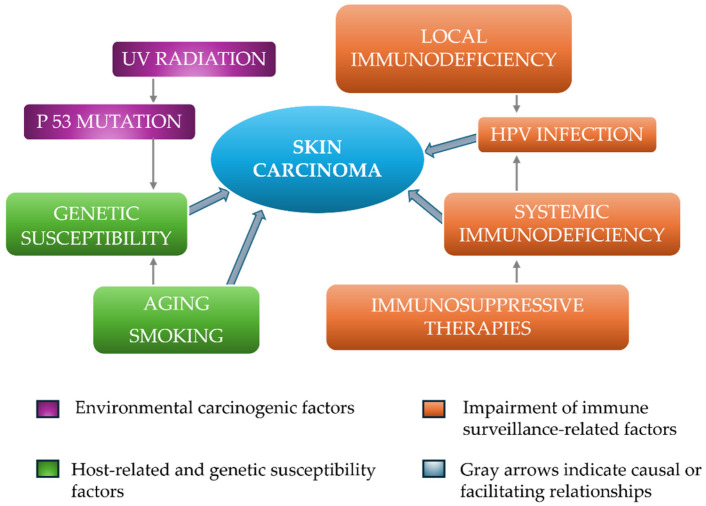
Multifactorial mechanisms contributing to skin carcinogenesis. APC: Antigen Presenting Cells. HPV: Human Papilloma Virus. UV: Ultraviolet (Rays). This schematic diagram illustrates the main environmental, genetic, and immunological factors involved in the development of skin carcinomas. Ultraviolet (UV) radiation represents a major environmental carcinogen and promotes DNA damage, including mutations in the tumor protein p53 (TP53), which contributes to malignant transformation of keratinocytes. Additional host-related factors such as genetic susceptibility, aging, and smoking may further enhance carcinogenic processes. Impairment of immune surveillance plays a critical role in tumor development: local immune deficiency can facilitate the persistence of oncogenic viral infections such as human papillomavirus (HPV), while systemic immunodeficiency—commonly associated with immunosuppressive therapies—reduces the body’s ability to eliminate transformed cells. The convergence of these environmental, host, and immune-related mechanisms ultimately promotes the development of skin carcinomas. Arrows indicate causal or facilitating relationships between contributing factors.

The development of cutaneous malignancies in transplant recipients is influenced by multiple factors (Figure 1). Both the duration and dose of immunosuppressive therapy are closely associated with increased incidence, with longer and more intensive regimens correlating with higher risk [4,21]. Environmental exposure, particularly cumulative ultraviolet (UV) radiation in fair-skinned individuals, contributes significantly [22]. Among transplant patients, those with lighter skin tones have a much greater likelihood of developing skin carcinoma than those with darker complexions [23].

Genetic susceptibility, such as certain HLA types, and oncogenic viral infections—including human herpesvirus 8 (HHV-8) and Merkel cell polyomavirus—further increase the likelihood of developing iatrogenic Kaposi’s sarcoma or Merkel cell carcinoma. The role of HPV in the development of skin cancers remains unclear in the literature. However, in a study by Harwood et al. HPV DNA was found in 33/40 SCCs and in 18/24 of BCCs of immunosuppressed patients [24].

### 3.2. Immunosuppressive Regimens and Oncologic Impact

#### 3.2.1. Calcineurin Inhibitors, Corticosteroids, and Azathioprine

Calcineurin inhibitors (cyclosporine and tacrolimus), corticosteroids, and azathioprine are associated with pro-oncogenic effects [14,25]. These include impaired immune surveillance, increased expression of Transforming Growth Factor beta (TGF-β), higher susceptibility to DNA damage, and resistance of tumor cells to apoptosis. 

Combination therapies, such as cyclosporine combined with azathioprine and corticosteroids, are linked to a significantly higher risk of cutaneous malignancy compared with less intensive regimens. Indeed, in a retrospective study conducted on approximately 300 patients, an increase in the annual incidence of NMSC from 29/1000 to 48/1000 cases was observed, along with an earlier onset of the disease [26]. This was shown by comparing two groups treated with immunosuppressive therapies for renal transplant: in the first group, patients received therapy with azathioprine and prednisone, whereas in the second group, cyclosporine was added to these two treatments.

#### 3.2.2. Mycophenolate Mofetil (MMF)

In contrast, mycophenolate mofetil exhibits a more favorable oncologic profile [27]. Its use has been associated with a limited risk of non-melanoma skin cancer and post-transplant lymphoproliferative disorders, making it a relatively safer option in long-term immunosuppressive protocols.

Indeed, according to the study by Yusuf et al., which included 941 kidney transplant recipients, MMF-based immunosuppression was more strongly associated with the development of BCC than SCC in both males and females. Among affected male patients, BCC accounted for 41.7% of cases (5 cases) compared to 25% for SCC (3 cases). Similarly, in female patients, BCC represented 50% of cases (5 cases), whereas SCC accounted for 20% (2 cases) [28].

Although most available evidence derives from kidney and other solid organ transplantation, these observations remain relevant for vascularized composite allotransplantation. Immunosuppressive regimens used in facial and hand VCA are largely extrapolated from solid organ transplantation protocols, and therefore, similar oncologic considerations may apply [1,13].

#### 3.2.3. mTOR Inhibitors (Sirolimus, Everolimus)

Emerging evidence supports the potential protective role of mTOR inhibitors such as sirolimus and everolimus. These agents have demonstrated anti-tumoral effects in certain studies, reducing the incidence of NMSC, and are increasingly being integrated into both solid organ and facial/hand VCA protocols, either as part of maintenance therapy or as a conversion strategy in high-risk patients [29,30].

Indeed, in a study of 95 living and deceased donor kidney transplant recipients followed for a median of 7.3 years, the hazard ratio [HR] of developing non melanoma skin cancer was 0.2 (95% CI 0.02, 0.92) in the everolimus-treated group, 0.34 (95% CI 0.13, 0.91) in the group treated with MPA (mycophenolate sodium) plus everolimus [31].

In a meta-analysis by Wolf et al., including 20 studies with more than 7000 patients, combined therapy with sirolimus and calcineurin inhibitors was shown to have the greatest antineoplastic effect, with a relative risk of 0.23 compared with other regimens using calcineurin inhibitors alone, sirolimus alone, or everolimus alone. The authors also attributed this finding to the fact that only two studies reported the overall incidence of malignancies across these therapeutic regimens [32].

Furthermore, mTOR inhibitors have been shown to act particularly by reducing the incidence of cutaneous tumors, while also exerting a protective effect against other malignancies.

These findings may also have important implications for facial and hand VCA, where mTOR inhibitors are increasingly considered as part of immunosuppressive minimization strategies aimed at reducing long-term oncologic risk.

#### 3.2.4. Biological Agents 

Lymphocyte-depleting antibodies, like Thymoglobulin, are associated with an increased risk of skin cancer or other malignancies [33].

According to a study by Puttarajappa et al., among 1350 kidney transplant recipients, malignancies excluding non-melanoma skin cancer occurred in 5.4% of patients treated with thymoglobulin (1.09 per 100 patient-years), compared with 2.8% of those receiving alemtuzumab (0.74 per 100 patient-years) and 3.3% of patients who received no induction therapy (0.66 per 100 patient-years), indicating a higher post-transplant malignancy risk associated with thymoglobulin [33].

Although these data cannot be directly applied to the assessment of cutaneous oncologic risk—given the exclusion of non-melanoma skin cancers—they nonetheless demonstrate an overall increase in malignancy risk under thymoglobulin. This broader oncologic susceptibility may therefore still be considered a relevant factor when evaluating the potential cancer risk profile associated with this immunosuppressive agent [34].

### 3.3. Immunosuppression in Facial and Hand VCA

In facial and hand vascularized composite allotransplantation, induction therapy most commonly includes thymoglobulin and high-dose methylprednisolone, with occasional use of basiliximab or alemtuzumab. Maintenance therapy typically relies on triple-drug regimens consisting of tacrolimus, mycophenolate mofetil, and corticosteroids, largely extrapolated from solid organ transplantation [1,13] (Table 1). However, vascularized composite allotransplants exhibit unique immunological characteristics, particularly related to the skin component, which harbors a high density of antigen-presenting cells and confers increased graft antigenicity. This results in persistent alloimmune stimulation and a propensity for the development of donor-specific antibodies, often necessitating intensified and prolonged immunosuppressive therapy compared with solid organ transplantation [35].

Despite these protocols, the incidence of malignancy reported in facial VCA (fVCA) recipients is approximately 10–11% [1]. This estimate is derived from the 2024 literature review by Hadjiandreou et al., which analyzed 46 reported face transplant cases, the majority of which were treated with immunosuppressive regimens analogous to those used in solid organ transplantation, namely mycophenolate mofetil, tacrolimus, and corticosteroids. Notably, the observed malignancy rate appears comparable to that reported in solid organ transplant recipients. Similarly, the reported incidence of malignancy following hand transplantation is approximately 5%, based on a study by Milek et al. analyzing 39 hand transplant recipients [36].

**Table 1:** Reported oncologic complications in fVCA and hand VCA.

Patient	Year of Transplant	Malignancy	NMSC	Months from Transplant	Immunotherapy Induction	Immunotherapy Maintenance	Other Complications	Death
Facial VCA (1)	2005 [1]	Yes	Lung SCC Cervical SCC HPV+ BCC of the face * [2,37,38]	132 (lung SCC) 50 (cervical HPV) 72 (BCC)	Thymoglobulin	Tacrolimus MMF Steroids	NR	Yes
Facial VCA (7)	2009 [1,38]	Yes	No hCC	65	Thymoglobulin	MMF MP Tacrolimus	Contestual bilateral hand transplant, acute rejection of both hands	Yes
Facial VCA (9)	2009 [39]	Unclear	No Pseudosarcomatous spindle-cell postsurgical nodule was removed from the base of the tongue (transplanted)	11	Anti-CD25 Basiliximab	Tacrolimus MMF Steroids Tacrolimus → Sirolimus after 11 months	HIV+	No
Facial VCA (10)	2009 [1]	Yes	No Large cell Epstein–Barr virus-associated B-cell lymphoma Hepatic post-transplant smooth muscle tumour	6 (EBV-associated B-cell lymphoma) 24 (hepatic post-transplant smooth muscle tumour)	NR	NR	Kidney transplant required for hepatic post-transplant smooth muscle tumour FRFF for lesions at the oral commissure due to chronic rejection	No
Facial VCA (29)	2013 [1,2,37,40]	Yes	Yes SCC (upper/lower extremity)	5	Thymoglobulin Steroids	Tacrolimus	Preauricular large B-cell lymphoma (on transplanted skin)	Yes
Bilateral hand VCA	2006 [41]	Yes	Yes BCC right nasal ala	12	Alemtuzumab	Tacrolimus MMF Prednisone Tacrolimus → Sirolimus (190 day)	NR	No
Monolateral hand VCA	2006 [37,42,43,44]	Yes	No Lymphoproliferative disease	23	Alemtuzumab	Tacrolimus MMF	3 rejection episodes	No

NR: Not reported; VCA = vascularized composite allotransplantation; MMF = mycophenolate mofetil; MP = methylprednisolone; SCC = squamous cell carcinoma; BCC = basal cell carcinoma; EBV = Epstein–Barr virus; hCC = hepatocellular carcinoma; → = switched to.* reported only by Kanitakis et al. [2] but not reported by any other major reporting registries by Petruzzo et al. [37] and Diep et al. [38].

## 4. Discussion

Cutaneous malignancies represent the most frequently encountered post-transplant neoplasms and remain a major long-term complication of chronic immunosuppressive therapy [4]. While the incidence and behavior of NMSC, particularly SCC and BCC, are well documented in solid organ transplantation [45], the evidence available for vascularized composite allotransplantation (VCA) remains limited by heterogeneity of reporting, small cohort sizes, and incomplete oncologic characterization. In many publications, although oncologic complications are reported, it is often difficult to identify the associated data, the timing of onset, and the specific types of malignancies. Moreover, most available evidence derives from case series or outcomes reported at different follow-up intervals after the same transplant procedure. 

Consequently, the reported incidences—approximately 10–11% in facial VCA and around 5% in hand VCA [1]—likely underestimate the true burden of disease.

Among the 46 facial transplant procedures reported in the literature, long-term oncologic complications and reported cases of malignancy have been described in five cases, as summarized in Table 1 [40,46,47,48,49]. 

However, this figure should be interpreted with caution. In the remaining cases, the absence of reported malignancies does not necessarily indicate that cutaneous tumors did not occur; rather, oncologic outcomes are often incompletely described or not systematically reported. 

Consequently, reliable comparisons between patients who developed malignancies and those who did not are difficult. The literature lacks consistent reporting of both oncologic events and detailed immunologic parameters, making it unclear whether patients who develop cutaneous malignancies have a distinct immunologic profile. 

Future studies integrating oncologic outcomes with detailed immunologic monitoring may help clarify whether differences in immune status contribute to cancer susceptibility in VCA recipients.

Another important consideration is the variability in induction and maintenance immunosuppression. Although treatment protocols are generally adapted from solid-organ transplantation, not all patients received the same agents, dosages, or treatment durations. Because both the type and cumulative exposure to immunosuppression influence post-transplant malignancy risk, well-designed randomized studies are needed to better define the oncologic safety profile of facial transplantation.

The creation of dedicated, standardized multicenter databases is therefore essential. Existing registries, including the SRTR (Scientific Registry of Transplant Recipients) and IRHCTT (International Registry on Hand Composite Tissue Transplantation) [37,50,51], although valuable, often lack critical variables such as tumor histology, site of onset (transplanted versus native skin), donor and recipient phototype, UV-exposure history, immunosuppressive regimen, and details on rejection episodes and rescue therapy. Improved registry completeness and long-term dermatologic follow-up would enable more accurate assessment of tumor incidence, latency, and risk factors. 

Primary prevention remains a cornerstone of management: rigorous photoprotection, patient education, regular dermatologic surveillance, and early immunosuppressive modulation—particularly the introduction of mTOR inhibitors, which may reduce NMSC risk [30,32,52]—should be implemented systematically, especially given that facial and hand VCA involve highly immunogenic, chronically UV-exposed tissues requiring prolonged and often intensified immunosuppression [10,35]

A second, more exploratory consideration concerns the potential biologic and oncologic implications of transplanting donor skin—an organ with its own immunologic identity, photobiologic history, and cumulative DNA damage—into a recipient maintained under chronic immunosuppression [11]. These reflections should be regarded as hypothesis-generating observations, as direct evidence addressing these questions in vascularized composite allotransplantation remains extremely limited.

Unlike internal organs, skin retains intrinsic characteristics such as phototype-related UV susceptibility, melanocyte function, oncogenic viral exposure, and pre-existing mutational signatures. As a result, the donor integument may exhibit a carcinogenic risk profile distinct from that of the recipient’s native skin. 

It remains unclear whether donor phototype influences graft malignancy risk. A lighter-phototype graft may be more vulnerable in a darker-phototype recipient, whereas darker skin may confer relative protection in a lighter-phototype recipient.

Likewise, no study has determined whether cutaneous malignancies arising in VCA originate from donor or recipient keratinocytes. Furthermore, it remains unclear whether rejection episodes, inflammatory injury, and lymphocyte-depleting therapies collectively predispose donor skin to neoplastic transformation. 

The current literature does not distinguish whether tumors in VCA patients arise within the transplanted skin or in the recipient’s native integument, and no systematic comparison exists between these two compartments. The following considerations should be interpreted as hypothesis-generating observations, as direct evidence addressing these questions in VCA remains extremely limited. This represents a significant gap that must be addressed to define whether transplanted skin demonstrates increased susceptibility, a risk comparable to native skin under immunosuppression, or unique patterns of transformation driven by donor–recipient immunogenetic interactions. Future studies incorporating donor–recipient genetic analysis and long-term dermatologic surveillance will be necessary to clarify these questions and determine whether transplanted skin exhibits a distinct carcinogenic risk profile.

Finally, these considerations must be contextualized within the ethical framework specific to VCA. Unlike solid organ transplantation, where immunosuppression is justified by its life-saving role, fVCA exposes otherwise stable patients to a lifelong risk of malignancy in exchange for functional and psychosocial improvement. This creates a distinct risk–benefit balance that requires careful oncologic consideration. In kidney transplantation, where immunosuppression is essential for survival, the increased incidence of malignancy is well-documented and generally accepted because of the life-preserving nature of the procedure.

In contrast, fVCA requires exposing patients to comparable or even higher levels of immunosuppression despite the absence of a vital indication [35]. This raises questions regarding acceptable risk, informed consent, and the obligation to minimize harm while maximizing functional and psychosocial benefit. Transparent communication of long-term carcinogenic risks, judicious selection of immunosuppressive agents with lower oncogenic potential, and intensified dermatologic surveillance are therefore ethically indispensable. 

Moreover, rigorous preoperative patient selection is essential in candidates for facial transplantation, as a prior history of neoplasia constitutes an additional risk factor for the development of de novo malignancies or recurrence of previous tumors. 

Comprehensive oncologic screening prior to transplantation is therefore critical to minimize these risks and to ensure that this treatment is undertaken only under conditions that uphold clinical safety and ethical responsibility [53]. As long-term data continue to evolve, ongoing reassessment of the risk–benefit ratio will be crucial to ensure that VCA is offered within ethically sustainable boundaries.

## 5. Conclusions

Cutaneous malignancies represent a major long-term complication of immunosuppressive therapy in transplant recipients. In facial vascularized composite allotransplantation (fVCA), the oncologic risk has not been specifically studied, as immunosuppressive regimens are largely extrapolated from solid organ transplantation. While the intrinsic risk may not be inherently higher in facial grafts, the greater immunogenicity of skin and mucosa often necessitates combined and prolonged immunosuppressive therapy, which may increase long-term cancer risk.

Careful patient monitoring, rigorous photoprotection, and consideration of less oncogenic regimens, including mTOR inhibitors, are recommended to minimize risk. Further research, including long-term prospective studies and the establishment of dedicated registries, is essential to clarify the true incidence of cutaneous malignancies and to optimize immunosuppressive strategies in facial VCA, balancing graft survival with patient safety and quality of life.

## Data Availability

No new data were created or analyzed in this study.
